# Supplementary material for: Prevalence of Flavescence Dorée Phytoplasma-Infected Scaphoideus titanus in Different Vineyard Agroecosystems of Northwestern Italy
Source: Insects. 2020 May 13;11(5):301. doi: 10.3390/insects11050301 (PMC7290324; doi:10.3390/insects11050301)
Supplement: Supplementary file 1 [file insects-11-00301-s001.zip › TableS2_Rev.docx]

**Table 2**. Numbers of *Scaphoideus titanus* adults collected by yellow sticky traps inside and outside the seven investigated vineyards. Time period: A, July 10^th^ to 31^th^; B, July 31^st^ to August 21^th^; C, August 21^st^ to September 9^th^. Acronyms: AT, Asti; CI, Cisterna d’Asti; CR, Castel Rocchero; LM, La Morra; MO, Montà d’Alba; PA, Paderna; PC, Portacomaro. NC: not collected

| **Site** | **Inside** | | | **Infected/Tested (inside)** | | | **Outside** | | | **Infected/Tested (outside)** | | |
| --- | --- | --- | --- | --- | --- | --- | --- | --- | --- | --- | --- | --- |
|  | **A** | **B** | **C** | **A** | **B** | **C** | **A** | **B** | **C** | **A** | **B** | **C** |
| **AT** | 11.80 ± 6.70 | 4.30 ± 2.90 | 0.75 ± 1.00 | 4/47 | 29/54 | 1/2 | 113.80 ± 118.60 | 55.80 ± 61.20 | 6.30 ± 5.00 | 5/28 | 8/30 | 12/30 |
| **CI** | 17.33 ± 17.33 | 7.00 ± 6.02 | 2.56 ± 4.45 | 43/108 | 42/60 | 5/6 | 23.20 ± 37.50 | 12.60 ± 14.50 | 6.60 ± 5.50 | 11/49 | 51/74 | 32/33 |
| **CR** | 72.30 ± 89.40 | 21.30 ± 11.50 | 0.30 ± 0.60 | 18/46 | 9/30 | 1/2 | NC | NC | NC | NC | NC | NC |
| **LM** | 74.60 ± 71.40 | 45.00 ± 32.00 | 16.80 ± 16.90 | 3/48 | 2/27 | 3/43 | 142.00 ± 98.20 | 90.40 ± 78.40 | 67.60 ± 56.00 | 15/36 | 44/71 | 28/41 |
| **MO** | 35.00 ± 14.80 | 6.33 ± 5.51 | 3.33 ± 3.51 | 23/56 | 15/46^a^ | 9/14 | 30.20 ± 29.20 | 23.80 ± 15.40 | 1.70 ± 2.90 | 31/37 | 16/58 | 10/19 ^a^ |
| **PA** | 33.30 ± 5.60 | 15.00 ± 3.90 | 3.50 ± 1.70 | 3/59 | 3/40 | 2/35 | 23.10 ± 21.30 | 22.00 ± 26.90 | 10.80 ± 13.60 | 1/19 | 16/68 | 1/24 |
| **PC** | 25.00 ± 3.00 | 15.30 ± 8.50 | 10.00 ± 9.90 | 22/38 | 8/32 | 12/26 | 23.50 ± 21.30 | 22.00 ± 26.88 | 10.75 ± 13.60 | 51/88 ^b^ | 27/57 ^b^ | 27/56 ^b^ |

^a^tested leafhoppers were collected both by yellow sticky traps and sweeping net

^b^tested leafhoppers were collected by sweeping net
